# The Treatment of Tinea Capitis

**Published:** 1899-12-09

**Authors:** 


### THE TREATMENT OF TINEA CAPITIS.

In a clinical lecture on Tinea Capitis, Dr. Shoemaker,[1] of Philadelphia, recommends the use of an ointment containing oleate of copper, one part of the latter to four, six, or eight of the ointment base. He does not recommend shaving, epilation, or forcible measures of any kind, for the reasons that they produce pain and not infrequently make matters worse. The same objections, he says, apply equally to powerful chemical solutions. In the particular case which was the subject of his lecture he ordered an ointment containing 20 per cent. each of oleate of mercury and oil of cade, with olive oil as an excipient, and as the child was of weakly habit he gave him iodide of iron and cod liver oil.

[1] Med. Bulletin, Nov., 1899.

